# Genetic Mapping of Loci Controlling Grain Size in Central Asian Wheat Accessions Through GWAS and QTL Mapping

**DOI:** 10.3390/plants15213361

**Published:** 2026-10-29

**Authors:** Kanat Yermekbayev, Madi Shoken, Zhuldyz Sartbayeva, Aibek Abduakassov, Makpal Bauyrzhan, Symbat Gabitkyzy, Hakan Ozkan, Bakyt Ainebekova

**Affiliations:** 1 Institute of Biotechnology and Ecology, Zhetysu University named after I.Zhansugurov, Taldykorgan 040009, Kazakhstan; shoke7777777@gmail.com (M.S.); sartbayevazhuldyz@gmail.com (Z.S.); aibekabduakas@gmail.com (A.A.); makpalbaurjan05@gmail.com (M.B.); symbatgabitkyzy316@gmail.com (S.G.); 2 Institute of Plant Biology and Biotechnology, Almaty 050040, Kazakhstan; 3 Department of Field Crops, Faculty of Agriculture, University of Çukurova, Adana 01330, Türkiye; hozkan@cu.edu.tr; 4 State Key Laboratory of Wheat Improvement, College of Agronomy, Shandong Agricultural University, Daizong Street 61, Taian 271018, China; 5 Kazakh Scientific Research Institute of Agriculture and Plant Growing, Almalybak 040909, Kazakhstan; bakyt.alpisbay@gmail.com

**Keywords:** Central Asia, Central Asian commercial wheat accessions, RILs, GWAS, QTL mapping, grain length, grain width

## Abstract

Although grain size is an important trait in Central Asian (CA) wheat breeding and industry, it remains genetically uncharacterized. We conducted GWAS and QTL mapping using a commercial wheat diversity panel (DP) and segregating population (SP). No genetic or phenotypic variation in grain width (GW) was detected in the studied panel under the conditions examined. For grain length (GL), we identified 37 significant MTAs on chromosomes 1A, 1B, 1D, 2B, 2D, 3A, 3B, 3D, 4B, 5A, 5B, 5D, 6A, 6B, 7A, 7B and 7D. The MTA on chromosome 2D (TaGL2DMTA) was identified by three GWAS models simultaneously. Two loci controlling GL, on chromosomes 2D (*TaGL2DQTL)* and 3A (*TaGL3AQTL*), were identified from QTL analysis. Loci on chromosome 2D were detected by complementary GWAS and QTL. *TaGL2DMTA*, *TaGL2DQTL* and *TaGL3AQTL* accounted for 12.4–15.5%, 14.5–18.3%, and 24.4–28.2% of the phenotypic variation, and increased GL by 6.8% (*p* = 0.0009), 2.3% (*p* = 0.024) and 4.0% (*p* = 0.0001), respectively. When these were converted to KASP markers, GL was significantly different between effective and non-effective alleles for *TaGL2DMTA* (*p* = 0.001) and *TaGL3AQTL* (*p* < 0.003). The validation of *TaGL2DQTL* highlighted limited effective allele distribution (~3%) in the DP. GL was positively correlated with GWT and TGW in both DP (r = 0.166/*p* = 0.03 and 0.526/*p* = 0.001, respectively) and SP (r = 0.212/*p* = 0.08 and 0.640/*p* = 0.001, respectively). Despite seasonal variation in GL, heritability was high, with H^2^ = 0.91 for DP and 0.93 for SP, indicating high genetic stability. Overall, these findings provide new insights into the genetic control of grain size, and highlight the identified loci as promising targets for marker-assisted selection in CA wheat breeding.

## 1. Introduction

As a major cereal crop, providing approximately 18–20% of global dietary calories and protein, bread wheat (*Triticum aestivum* L.) plays a crucial role in global food security and sustainable food production [1,2]. Its role in Central Asian food security systems is strategic: any improvement in grain yield and quality has direct consequences on the lives of millions of people living in the region and beyond [3].

Kazakhstan is a major wheat producer and supplier in Central Asia. Almost 80% (8–10 M/t) of wheat produced in the Kazakh steppes is destined for Uzbekistan, Tajikistan, Kyrgyzstan, Turkmenistan and Afghanistan, regions with higher levels of food insecurity. At the same time, Kazakhstan ranks among the world’s top 15 largest wheat providers contributing to the global food security. Despite this regional and global significance, the Kazakh wheat sector faces significant challenges. There has been substantial fluctuation in the wheat growing area—declining from 15.3 M/ha in the 1990s to 8.7 M/ha in the 2000s before recovering to 12 M/ha in 2026—which has impacted overall production. Wheat has also become less profitable compared to legumes despite their limited local use. In addition, almost 40% of the gene pool originates from international breeding programs, while locally developed lines remain sensitive to environmental constraints, so that farmers are not fully satisfied. Moreover, there is a significant gap between actual yield and production potential. Finally, grain yield is climate-dependent and unstable. It is likely that the Kazakhstani wheat breeding system has reached the maximum yield capacity using conventional methods. Therefore, further yield improvement requires continued genetic progress, using locally adapted germplasm to sustain regional wheat production.

In genetic studies, two complementary approaches are widely used for mapping of complex traits in plants. Genome-wide association studies (GWAS) exploit phenotypic variation and historical recombination events within natural diversity panels, enabling high-resolution mapping without the constraints of biparental crosses. GWAS has been applied extensively to yield-related traits in wheat, including grain size, using high-density SNP arrays [4,5,6]. However, rare alleles and population-specific variation can be missed in large diversity panels. QTL mapping in biparental recombinant inbred line (RIL) populations complements GWAS by providing controlled segregation, higher statistical power for minor-effect loci, and direct identification of donor allele information that is immediately actionable in breeding programs [7,8]. Combining both approaches increases confidence in detected loci, and enables cross-validation of results [9].

Grain morphology is believed to be one of the most frequently selected traits during domestication: grains of wild wheat relatives tend to be small and round, but domestication has progressively increased diversity in grain architecture [10,11]. In wheat, grain size directly influences grain yield and weight, and also affects milling, baking performance, and end-use quality [12,13,14,15,16,17]. It is a complex, yet highly heritable trait controlled by several loci; interaction of these with the environment highlights the sensitivity of grain size to environmental variation [18,19,20,21,22]. GL and GW have distinct genetic components and are thus controlled independently [10,15].

To date, numerous GWAS and QTL studies have identified genomic regions associated with grain morphometric parameters across diverse wheat populations and environments [10,12,13,15,17,19,21]. In addition, QTLs that influence grain size have been repeatedly identified across environments over many years on several wheat chromosomes, including 1A, 2A, 2D, 3A, 3B, 4B, 6A, 6B, 7A and 7D [22,23,24,25]. Consequently, genome-guided approaches have become increasingly important for dissecting the genomic regions controlling grain size, and for identifying loci that can be exploited in marker-assisted breeding.

Grain size is an important trait in Central Asian wheat breeding and industry. Although molecular marker-assisted approaches to wheat improvement have gained traction in recent years [4,6,26], the genetic basis of grain architecture remains uncharacterized in wheat germplasm from Kazakhstan, the main wheat provider in CA. Therefore, the aim of this study was as follows: (i) To identify significant variations in grain size; (ii) to genetically map them through GWAS and QTL mapping; and (iii) to develop and validate breeder-friendly KASP markers. To identify genomic regions controlling grain size specific to Central Asian wheat, we used a diversity panel of 153 elite spring wheat accessions and an RIL population derived from the cross of ‘Kazakhstanskaya Rannespelaya’ × ‘Paragon’. GWAS was conducted using the Axiom 35K SNP array, while QTL mapping employed the 43K TaNGv1.1 array and the R/qtl2 framework [27,28,29]. Candidate genes were annotated based on the IWGSC RefSeq v1.0 reference assembly and characterized using open-source evidence-based gene discovery and expression resources [30,31]. The outcomes are intended to support marker-assisted selection in CA wheat breeding programs and to contribute to the broader understanding of grain size genetics in hexaploid wheat.

## 2. Results

### 2.1. Phenotypic Variation and Correlation Analysis

**Figure 1:**
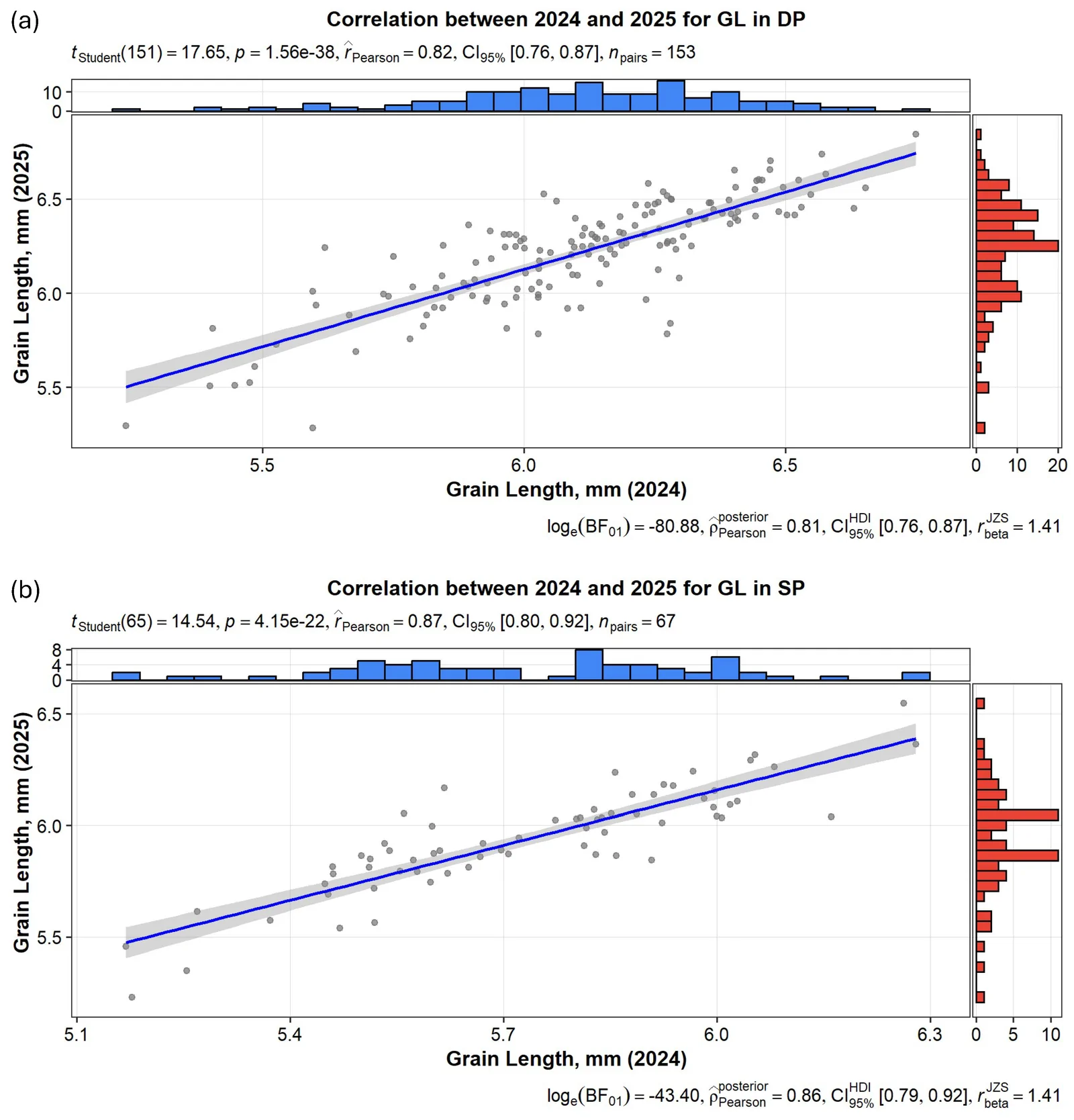
Correlation between GL measurements between growing seasons for (**a**) DP and (**b**) SP. Blue line: linear regression with 95% confidence interval highlighted in grey shading. Histograms show trait distribution in each growing season.

Bartlett’s test revealed no significant difference in variance between growing seasons (2024 and 2025) for either DP (K^2^ = 0.122, *p* = 0.727) or SP (K^2^ = 0.302, *p* = 0.583). Variation in grain length was significant in both the studied populations DP and SP (*p* = 0.001), but variation in grain width was not significant, indicating limited genetic diversity for this trait in Kazakh wheat germplasm. Pearson correlation analysis revealed a strong positive correlation between the two seasons in DP and SP (*p* = 0.001). The correlation coefficient for DP was *r* = 0.82, and for SP it was *r* = 0.87, indicating high consistency in GL across seasons (Figure 1).

**Figure 2:**
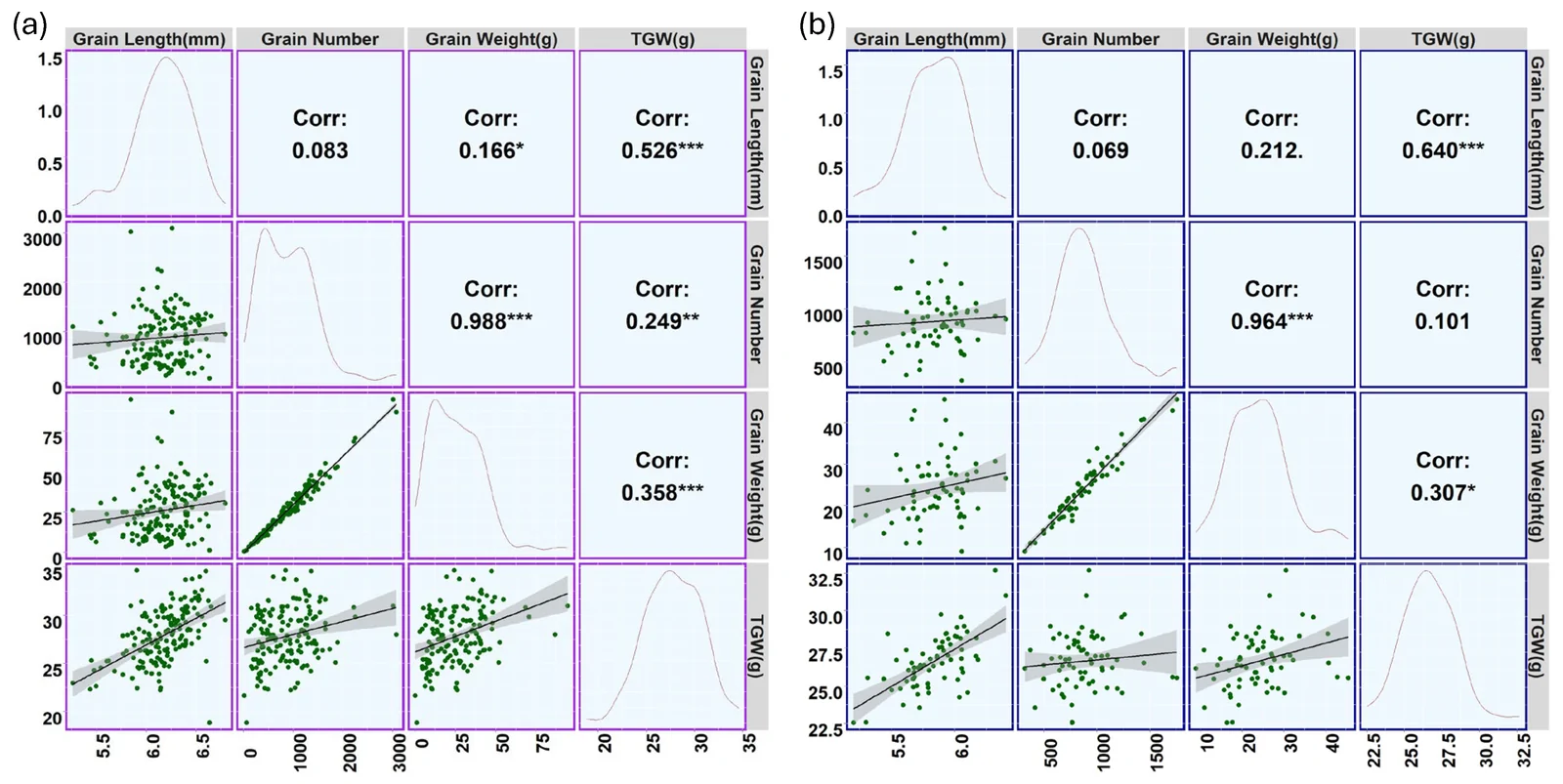
(**a**) Pairwise correlations among grain length (GL), grain number per row (GN), grain weight per row (GWT), and thousand grain weight (TGW) in DP. (**b**) Pairwise correlations among GL, GN, GWT, and TGW in SP. Diagonal panels show trait distributions by year; lower left panels show scatter plots with fitted regression lines; upper right panels report overall Pearson correlation coefficients. Significance codes: *** *p* < 0.001, ** *p* < 0.01, * *p* < 0.05.

Correlation analysis among grain morphological traits revealed a positive but non-significant association between GL (mm) and GN (per row) (*r* = 0.083, *p* = 0.30) in DP. However, GL was significantly positively correlated with GWT (per row, g) (*r* = 0.166, *p* = 0.03) and TGW (g) (*r* = 0.526, *p* < 0.001) in DP (Figure 2a). As with DP, GL was in a weak non-significant relationship with GN (per row) (r = 0.069, *p* = 0.58), while its association with GWT (per row, g) (*r* = 0.212, *p* =0.08) was close to significant in SP. In contrast, the correlation between GL and TGW (g) (*r* = 0.640, *p* < 0.001) was significant (Figure 2b). Despite seasonal variation in GL, heritability was high (H^2^ = 0.91 in the DP and 0.93 in the SP; r = 2 replicates) (Table 1). Genotypic variance (σ^2^G) was 0.067 in the DP and 0.05 in the SP, and residual variance (σ^2^ε) was 0.014 and 0.007, respectively. Genotype effects were significant in both populations (*p* < 0.001).

### 2.2. Population Structure

**Figure 3:**
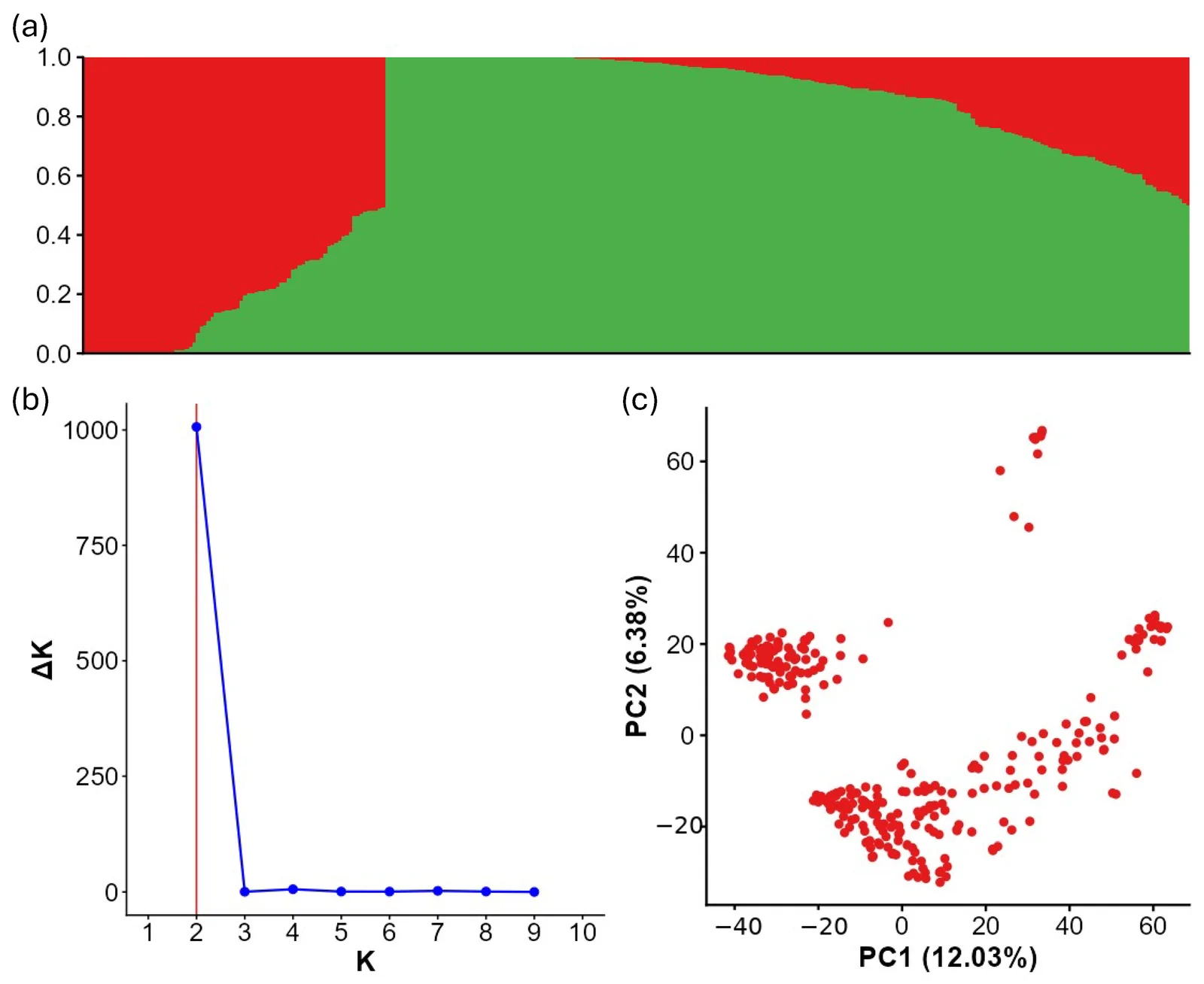
Population structure and principal component analysis of the DP. (**a**) STRUCTURE bar plot at the optimal K = 2; each vertical bar represents one accession, with colors indicating probability of membership for each subpopulation. (**b**) Delta K plot used to determine the optimal number of subpopulations; the peak at K = 2 indicates the most likely population structure. (**c**) Principal component analysis (PCA) scatter plot.

Structure analysis identified two distinct subpopulations among the wheat genotypes analyzed (Figure 3a). Using the Evanno method for Delta K analysis, we observed a strong peak at K = 2 (ΔK = 1006.58). In contrast, the rest showed minimal Delta K values (Table 2) (Figure 3b), indicating the presence of two primary subpopulations. Furthermore, th8e PCA plot reinforced this finding through evident population stratification. The first principal component (PC1) accounted for 12.03% of the total genetic variance, while the second principal component (PC2) contributed 6.38% (Figure 3c). Notably, the distribution of genotypes in the PC1–PC2 scatter plot revealed clear clustering into two major groups, a finding in good alignment with the structure results.

### 2.3. Final Genetic Map and GWAS for Grain Length

**Figure 4:**
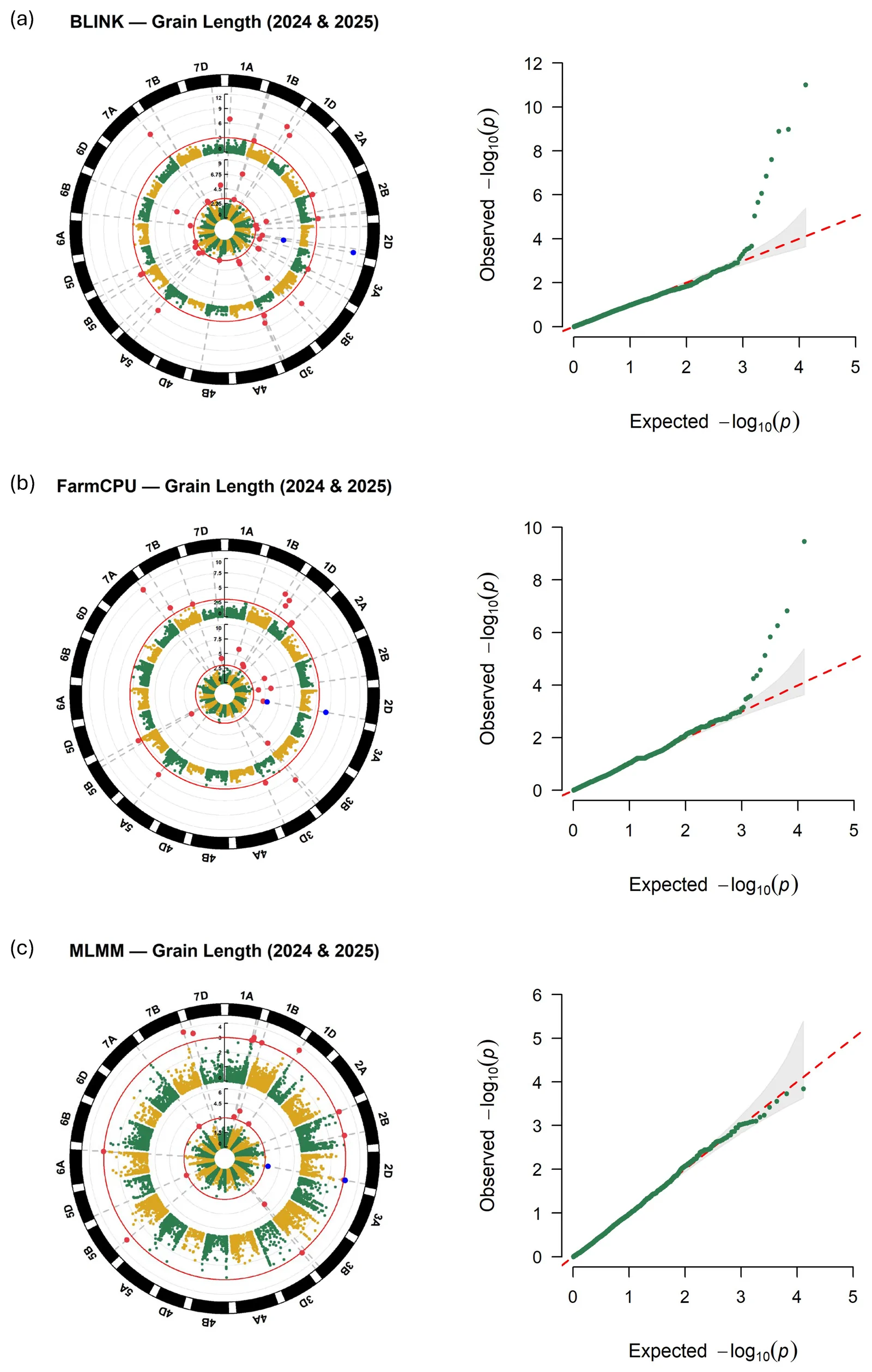
Circular Manhattan plots for GL across two growing seasons (2024: inner ring, 2025: outer ring) in the DP. (**a**) BLINK and Q-Q plot, (**b**) FarmCPU and Q-Q plot, (**c**) MLMM and Q-Q plot. Red dots indicate significant associations (−log_10_(*p*) ≥ 3). The blue dot marks the key stable MTA, identified through GWAS and QTL, linked to the AX-94457710 SNP marker on chromosome 2DL.

The initial dataset of diversity panel (DP) comprised 35K SNP markers. After filtering for missing data, minor allele frequency, and heterozygosity, 12,833 high-quality SNPs were retained for GWAS. Across the two growing seasons and three GWAS models (BLINK, FarmCPU, and MLMM), significant MTAs were detected on chromosomes 1A, 3A, 5A, 6A and 7A in the A genome; on chromosomes 1B, 2B, 3B, 4B, 5B, 6B and 7B in the B genome; and on chromosomes 1D, 2D, 3D, 5D and 7D in the D genome. Among these associations, robust MTA on chromosome 2D (*TaGL2DMTA*), linked to the AX-94457710 SNP marker, was consistently identified by all three GWAS models in the growing seasons of both 2024 and 2025, with log_10_(*p*) values of 5.41 and 6.16, respectively (Figure 4 and Table 3). Therefore, this locus was further validated through developing a KASP diagnostic marker. No MTA was identified for GW.

### 2.4. Final Physical Map and QTL Analysis for Grain Length

**Figure 5:**
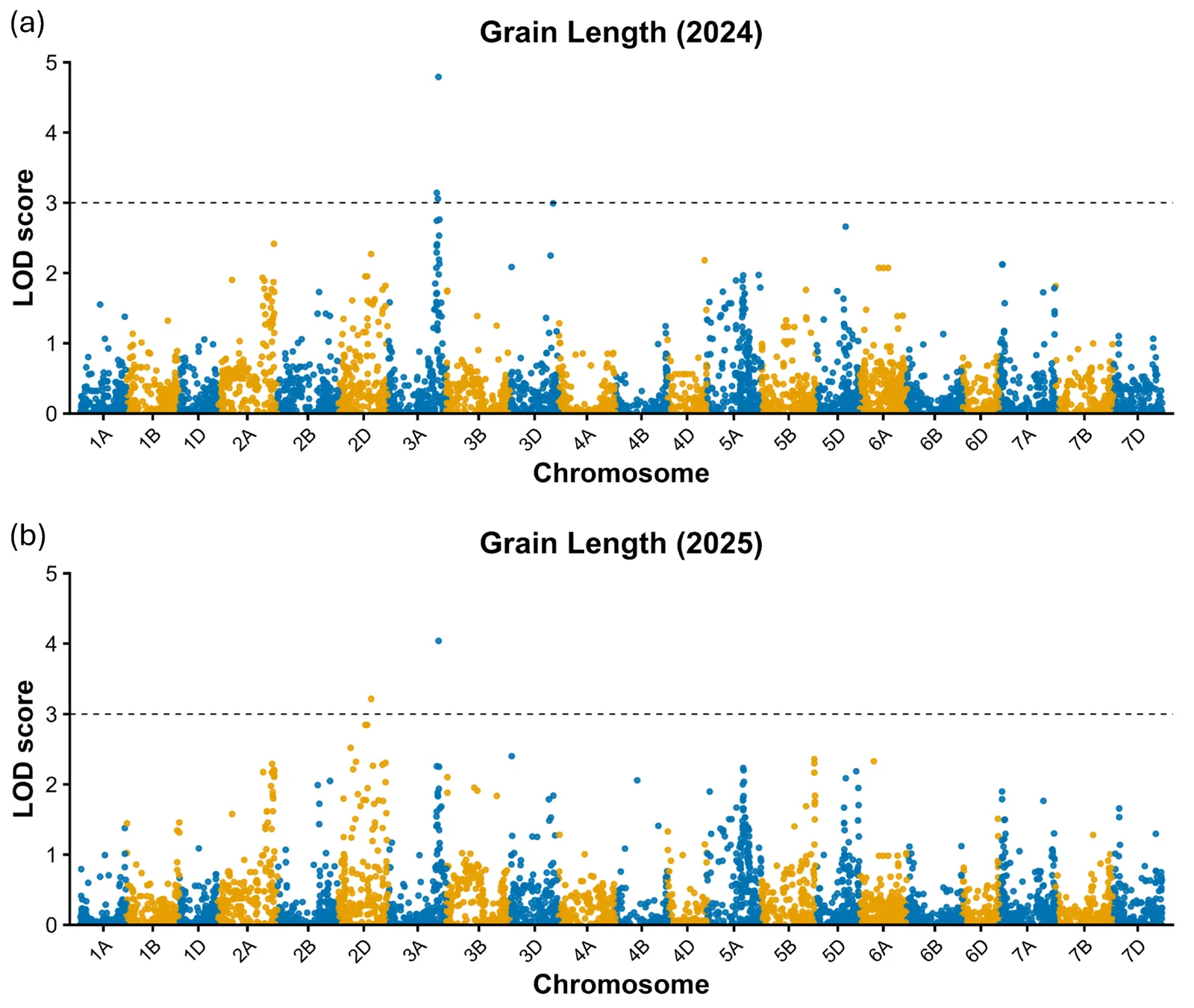
Manhattan plots for GL QTL analysis across two growing seasons: (**a**) 2024, (**b**) 2025. The dashed horizontal line indicates the significance threshold at LOD = 3.

The initial genetic dataset of SP consisted of 43K SNP markers. In Axiom Analysis Suite (v5.4), we retained highly polymorphic markers, reducing the set from 43,373 to 9202 SNPs. After conducting QC, the final genetic map of SP comprised of 5397 high-quality polymorphic SNP markers. We ordered markers and assigned chromosomes based on physical positions in IWGSC RefSeq v2.1. The final map contained 5397 SNPs on 21 chromosomes (130–340 per chromosome), with a span of 473–837 Mb per chromosome (14,025 Mb in total), and a mean density of 0.38 markers/Mb. The largest physical gap was 43.9 Mb on 4B. QTL intervals are reported in Mb between flanking markers. When assessed for possible clones in SP, the highest genetic similarity (93%) was identified between RILs KZRannxPar-01/KZRannxPar-05 and KZRannxPar-72/KZRannxPar-79. As with GWAS, the QTL analysis identified significant associations between the genotype and phenotype for GL but not for GW, again highlighting restricted genetic variation among the Central Asian wheat accessions for this trait. Significant QTLs for GL were identified on chromosomes 2DL and 3AL. The 3AL QTL (*TaGL3AQTL*) related to the SNP marker AX-643825530 (C/G) was consistent across the two growing seasons (2024 and 2025), but the association for 2DL QTL (*TaGL2DQTL*) in 2024 was not significant although the peak remained at an LOD score of 2.36 (Table 4 and Figure 5). However, the locus *TaGL2DQTL*, represented by the marker AX-643815838 (T/C), directly corresponded to the genomic region *TaGL2DMTA* identified from GWAS in the DP. The agreement between QTL mapping and GWAS results on chromosome 2DL and 3AL provided evidence for the possible involvement of this major region in grain length. Therefore, both QTLs were subjected to further genetic validation through KASP markers.

### 2.5. Development of KASP Markers and Their Validation

**Figure 6:**
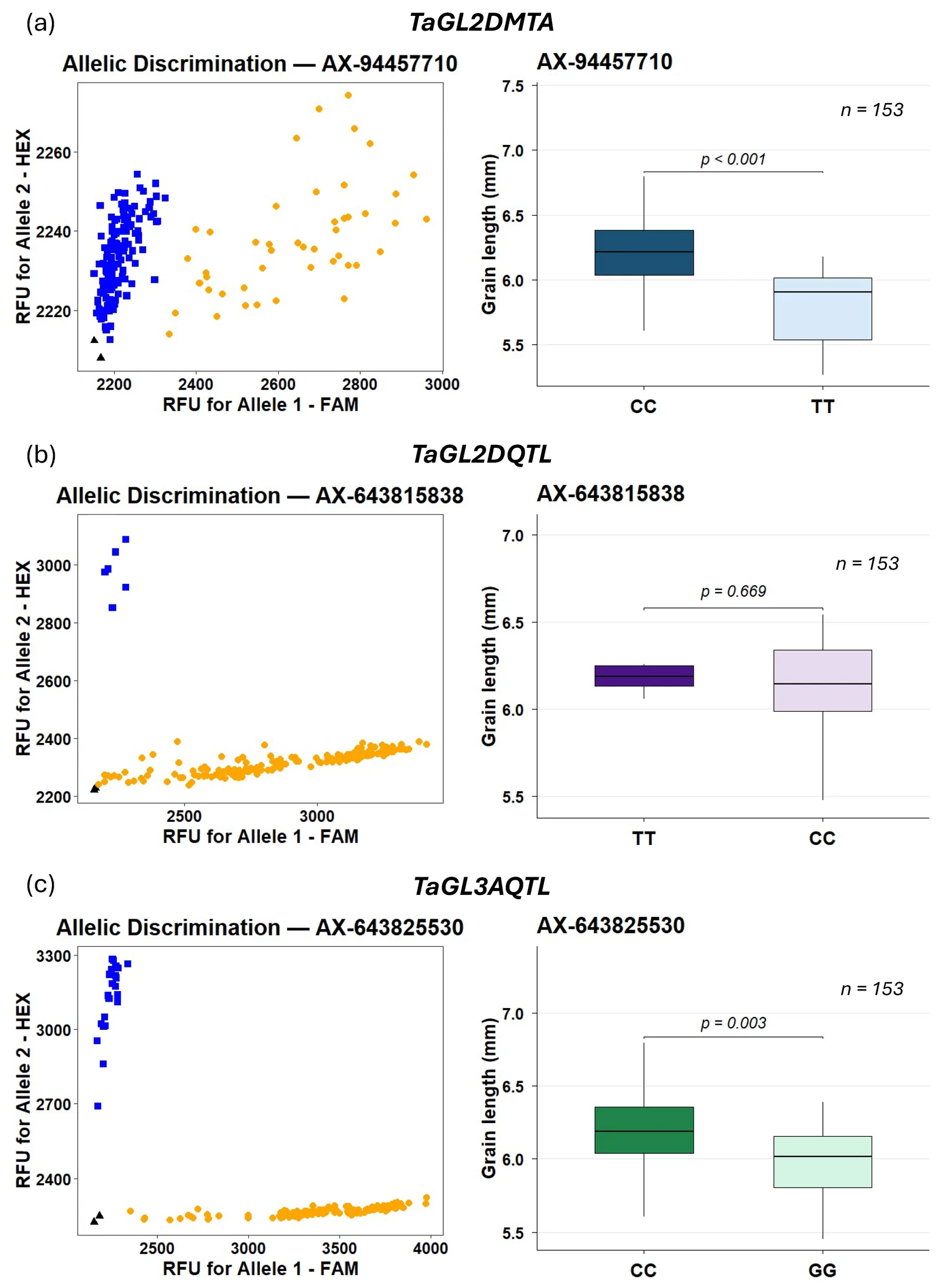
Allelic discrimination plots from GWAS and QTL studies for the three KASP markers associated with grain length in the Central Asian wheat panel, with corresponding box plots. (**a**–**c**) are related to *TaGL2DMTA*, *TaGL2DQTL* and *TaGL3AQTL*, respectively. Blue squares represent samples homozygous for Allele 1 (FAM-tailed), orange circles represent samples homozygous for Allele 2 (HEX-tailed), and black triangles indicate no-template controls (NTCs).

Diagnostic KASP markers were developed for the peak SNP marker, AX-94457710, that was significantly linked to the *TaGL2DMTA* locus identified from GWAS (Table 5). The *TaGL2DMTA* accounted for 12.4–15.5% of the phenotypic variation, and increased grain length by 6.8% (*p* = 0.0009). For significant associations identified from QTL analysis, we designed diagnostic KASP markers for peak SNPs related to *TaGL2DQTL* and *TaGL3AQTL* loci, located on chromosomes 2DL and 3AL, respectively (Table 5). The *TaGL2DQTL* loci (AX-643815838) on chromosome 2DL accounted for 14.5–18.3% of the phenotypic variation, and increased grain length by 2.3% (*p* = 0.024). In contrast, the *TaGL3AQTL* loci (AX-643825530) on chromosome 3AL accounted for 24.4–28.2% of the phenotypic variation, and increased grain length by 4.0% (*p* = 0.0001). The validation of the *TaGL2DMTA* locus-linked SNP marker (AX-94457710) on DP exhibited significant variation between effective and non-effective alleles (*p* = 0.001). However, further marker validation on other independent panels was required, as the DP was the panel in which the locus was initially identified. The results of validation for the *TaGL2DQTL* loci highlighted limited distribution of the effective allele in the DP, with only 3% of accessions carrying the positive allele at the loci. It is likely that, due to the limited sample size, the difference between effective and non-effective alleles was not statistically significant, although the mean of effective alleles was higher than that of non-effective alleles (*p* = 0.669). When the *TaGL3AQTL* locus (AX-643825530) was validated, the additive effect of the positive allele was significantly higher compared with that of the negative allele (*p* = 0.003) (Figure 6).

### 2.6. Effective Allele Distribution in DP

**Figure 7:**
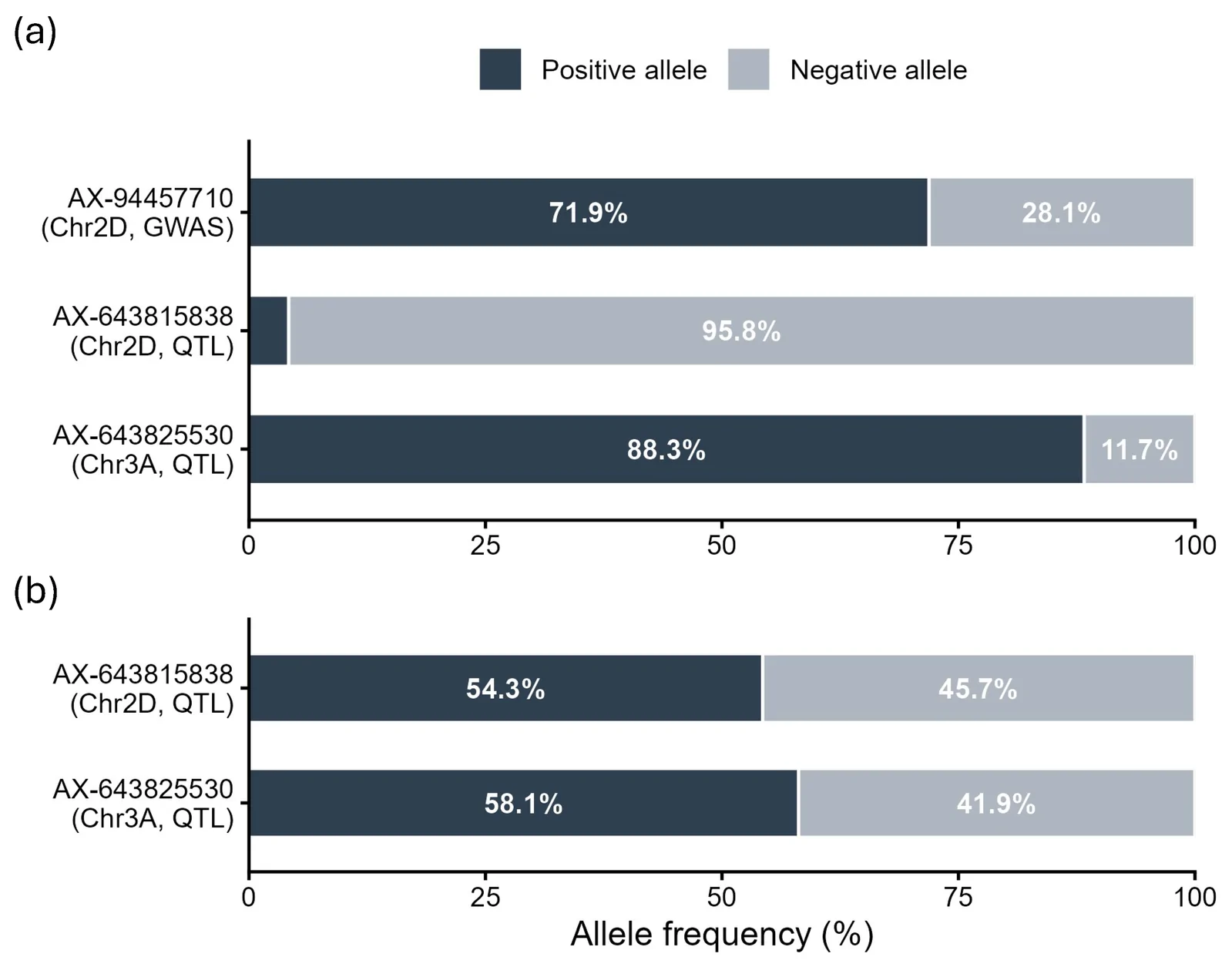
Effective allele frequency at the *TaGL2DMTA*, *TaGL2DQTL* and *TaGL3AQTL* loci in (**a**) DP, and (**b**) SP.

The distribution of favorable alleles was assessed by determining the number of effective alleles at the *TaGL2DMTA*, *TaGL2DQTL* and *TaGL3AQTL* loci in DP. Results showed that almost 72% of accessions in the Central Asian wheat panel possessed two copies of the effective allele C (homozygous CC) at the *TaGL2DMT*, indicating a high frequency of this favorable allele in the Central Asian wheat panel. In contrast, KASP genotyping revealed that most accessions carry a negative allele at the *TaGL2DQTL* locus, suggesting that this locus may represent a different genetic factor controlling grain length. In the case of *TaGL3AQTL*, we have seen that 88.3% of accessions in the DP were fixed for positive allele, demonstrating the effectiveness of the locus in marker-guided selection (Figure 7a). In the SP, both *TaGL2DQTL* and *TaGL3AQTL* loci showed balanced allele distribution; for example, the effective allele T (homozygous TT) at AX-643815838 (Chr2DL) was present at 54.3%, while the effective allele C (homozygous CC) at AX-643825530 (Chr3AL) was present at 58.1%, reflecting the RIL population.

### 2.7. Candidate Gene Annotation

**Figure 8:**
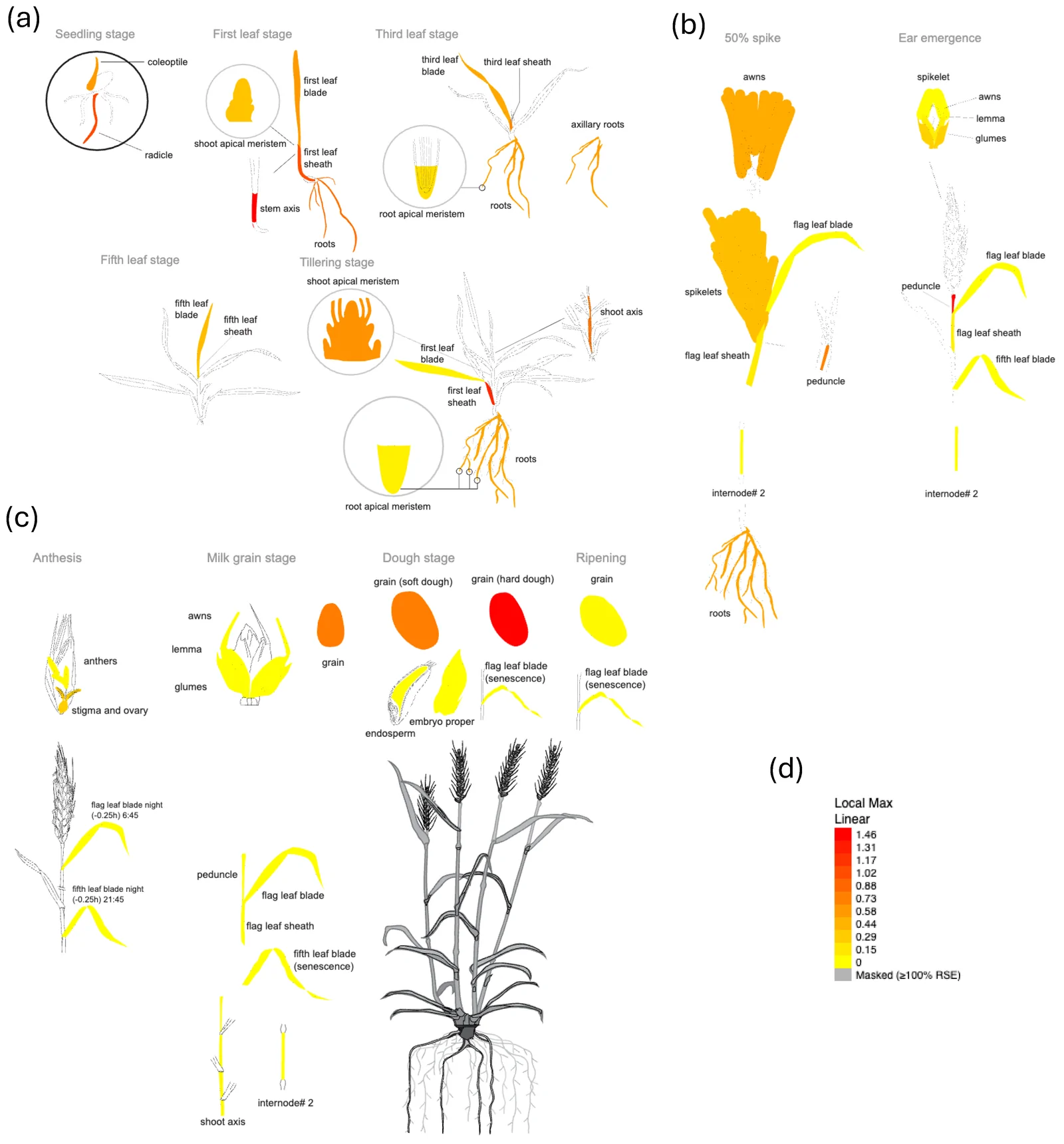
Expression profile of *TraesCS2D02G505700* in wheat based on RNA-seq data, showing the highest expression in developing embryos during grain development. The figure was generated using the eFP browser in ePlant (https://bar.utoronto.ca/eplant_wheat/, accessed on 25 October 2026). (**a**–**c**) show early, middle, and late developmental stages, respectively. (**d**) shows levels of expression.

**Figure 9:**
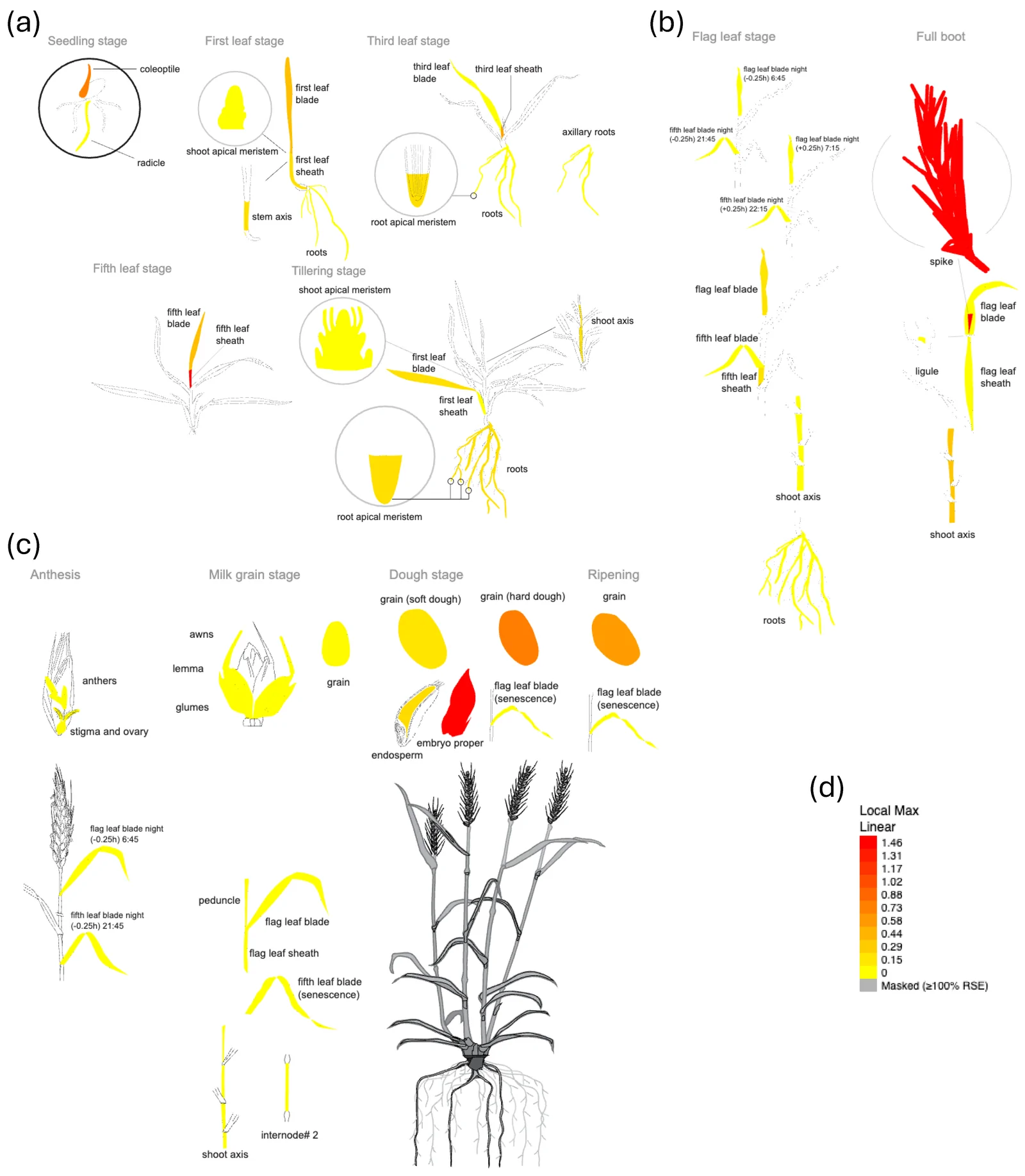
Expression profile of *TraesCS3A02G400500* in wheat based on RNA-seq data, showing the highest expression in developing embryos during grain development. The figure was generated using the eFP browser in ePlant (https://bar.utoronto.ca/eplant_wheat/, accessed on 25 October 2026). (**a**–**c**) show early, middle, and late developmental stages, respectively. (**d**) shows levels of expression.

Four high-confidence candidate genes (*TraesCS2D02G505700*, *TraesCS2D02G505900*, *TraesCS2D02G505800*, *TraesCS2D02G506000*) were found within the region of 30 kb upstream and downstream of the significant SNP marker (AX-94457710) identified from GWAS. Among these, the closest and most highly related gene was *TraesCS2D02G505700* [32,33,34]. This primary candidate gene was identified approximately 27.5 kb from the *TaGL2DMTA* locus (AX-94457710); this is highly expressed in the grain, according to transcriptomic analysis carried out on wheat (Figure 8) [31,35,36,37]. In addition, the *TraesCS2D02G505700* is located within the QTL flanking region of the *TaGL2DQTL* locus (AX-643815838). The co-localization of *TaGL2DMTA* and *TaGL2DQTL* at the distal end of chromosome 2DL highlights this region as a potential genomic region underlying variation in grain length in wheat, even though they might represent different loci. The nearest candidate genes associated with the *TaGL3AQTL* (AX-643825530) were identified at a location of approximately 150 kb upstream or downstream of the locus. This region contained four high-confidence genes (*TraesCS3A02G400200*, *TraesCS3A02G400300*, *TraesCS3A02G400400*, *TraesCS3A02G400500*) [32,33,34]. Among these, *TraesCS3A02G400500* was found to be highly expressed in the leaf sheath, spike, grain, and embryo, making it a promising candidate (Figure 9) [35,36].

## 3. Discussion

### 3.1. Phenotypic Variation, Heritability and Correlation

Larger grains are highly valued by those engaged in wheat breeding and industry in Central Asia. In addition, grain morphological traits are key factors influencing grain yields [13,18,19]. Therefore, this research study sought to identify genomic regions controlling grain size in the Central Asian wheat breeding pool through widely used genetic mapping methods. Grain length and width are major parameters of grain size. These parameters are genetically distinct traits, mostly controlled by independent genetic factors [10,15]. However, previous studies have also highlighted shared genomic regions with pleiotropic effects on grain length and width [12,38,39]. Longer grains seem to accumulate more starch and attain greater mass, and this suggests a causal link between grain size and weight, ultimately leading to increases in grain yield [10,13,40]. Productivity can be enhanced through improved germination and earlier vigor, both which are very important drivers of adaptation and yield in the semi-arid, harsh, and predominantly rain-fed wheat growing conditions of Central Asia [41,42,43]. Our results demonstrated significant variation in grain length among Central Asian commercial wheat accessions. In contrast, no genetic variation was detected for grain width, reflecting a narrower genetic basis for this trait in the CA commercial wheat panel. It is likely that GW exhibits lower genetic variation, compared to GL, in some wheat populations. Zhao et al. (2023) found that grain width possessed lower phenotypic variation and heritability than grain length in Chinese wheat lines [5]. These findings demonstrate that GW and GL are controlled by independent loci in the CA wheat panel. We also found that increased GL contributes to improved GWT and TGW, as evidenced by a significant positive correlation between these phenotypes across both panels (Figure 2a,b). These findings are in alignment with previous studies that reported moderate-to-strong correlations of GL with GWT and TGW [17,22]. Furthermore, the correlation between GL and GN was positive in both the DP and SP panels, albeit not statistically significant. This pattern is still favorable for breeding, given that GL and GN typically exhibit a trade-off relationship [44]. In general, grain morphometric traits exhibit high level of heritability [5,10,22]. Consistent with previous findings, we also observed a high heritability level of GL across seasons and populations, making this one of the most stable yield components that needs to be considered in Central Asian wheat breeding programs (Table 1).

### 3.2. Mapping of Genomic Regions Associated with GL and KASP Marker Development

In this study, we identified 37 significant MTAs on chromosomes 1A, 1B, 1D, 2B, 2D, 3A, 3B, 3D, 4B, 5A, 5B, 5D, 6A, 6B, 7A, 7B and 7D, based on three independent models from GWAS and two QTLs controlling GL from linkage mapping (Table 3 and Table 4). Among these, significant associations identified on the wheat chromosomes 2DL (*TaGL2DMTA/ TaGL2DQTL*) and 3AL (*TaGL3AQTL*) were transformed to, and validated as KASP markers, because the MTA on chromosome 2DL was identified across growing seasons (2024, 2025) by three GWAS models simultaneously (Table 5). Moreover, QTL analysis also identified a significant association close to this region on chromosome 2DL. Multiple earlier reports identified this region on chromosome 2DL as a key genomic region associated with GL [12,18,45,46,47,48,49]. Lyu et al. (2024) genetically mapped the transcription factor TabHLH489 to the chromosome 2DL, and identified that it regulates grain length through brassinosteroid and sugar signaling [49]. Importantly, this transcription factor is located 50 kb away from *TaGL2DMTA*. Moreover, Li et al. (2021) identified the stable *QGl.haaf-2DL.2/QTkw.haaf-2DL.2* locus around this region on the wheat 2DL chromosome [48]. The locus explained 13.6–19.8% of phenotypic variation, and co-localized with a TKW QTL. In this study, the *TaGL2DMTA* and *TaGL2DQTL* showed comparable effects on grain length, explaining 12.4–15.5% and 14.5–18.3% of the phenotypic variation, respectively, with the corresponding allelic effects associated with 6.8% (*p* = 0.0009) and 2.3% (*p* = 0.024) increases in grain length. Su et al. (2018) reported a pleiotropic cluster affecting kernel length and width on chromosome 2D [47]. The authors specifically noted that the region was close to the previously reported 2DL locus linked to the Xgwm539 SSR marker. In addition, the highly reproducible *qGL-2D* locus was identified on chromosome 2D using a highly diverse wheat collection [18]. Therefore, co-localization of *TaGL2DMTA* and *TaGL2DQTL* at the distal end of chromosome 2DL highlights this region as a potential genomic region underlying variation in grain length in CA wheat, and makes it a perfect target for marker-assisted selection. When *TaGL2DMTA* was transformed to KASP markers and validated on DP, it showed a significant association (*p* = 0.001) and demonstrated a robust linkage with GL (Figure 6). In contrast, while *TaGL2DQTL* amplified successfully, the effective allele showed limited distribution within the DP, a phenomenon best explained by statistical factors. In biparental populations, locus segregation typically follows a 1:1 ratio regardless of natural allele frequency, ensuring sufficient statistical power to detect an effect. Conversely, DP panels reflect natural allele frequency distributions; consequently, the sample size for a specific genotypic class may be not enough for reliable statistical analysis, increasing variance in effect estimation and reducing the statistical power of association detection. This aligns with the observation that the mean GL for samples carrying the effective allele was higher than for those carrying the non-effective allele, a trend consistent with the direction of the effect identified in the SP population. Thus, this result may not indicate an absence of the locus’s effect within the DP panel; it may instead indicate that the DP sample used does not allow reliable verification of its presence.

The *TaGL3AQTL* is the second locus which was consistently identified in QTL analysis across the two growing seasons of 2024 and 2025 in SP, highlighting its stability (Figure 5). GWAS also identified a significant MTA around the region of chromosome 3AL, underlying the importance of 3AL for grain length variation (Figure 4). Previous studies have shown that the wheat 3A chromosome harbors several loci underlying grain length variation [8,10,17,50,51]. Among these studies, Jardon et al. (2026) reported two QTL on the wheat 3A chromosome located at the physical coordinates of 623,038,468 and 618,164,627 [51]. These loci were approximately 24–29 Mb from *TaGL3AQTL*. Therefore, we hypothesize that *TaGL3AQTL* could be a putative novel locus. In another work, a strong QTL *Qgl.caas-3AL* was detected across 10 environments, and the locus explained 5.7–15.7% of GL variation [8]. In contrast, the *TaGL3AQTL* accounted for 24.4–28.2% of the phenotypic variation and increased grain length by 4.0% (*p* = 0.0001). Validation of the *TaGL3AQTL* in DP showed a strong positive effect (*p* = 0.003) (Figure 6). These findings improve our understanding of the genetic control of grain size, and highlight the identified loci as potential targets for marker-assisted selection in Central Asian wheat breeding.

### 3.3. Candidate Gene Prediction

In total, eight candidate genes were identified to be associated with grain biology in the proximity of genomic regions where the significant MTA and QTL SNP markers, related to the *TaGL2DMTA*, *TaGL2DQTL* and *TaGL3AQTL* loci, were identified. Among these, promising candidate genes, including *TraesCS2D02G505700* and *TraesCS3A02G400500*, were identified in association with the *TaGL2DMTA/QTL* and *TaGL3AQTL* regions, respectively. Both candidate genes showed significantly higher expression in developing grains, supporting their potential roles in GL variation. The candidate gene *TraesCS2D02G505700* encodes a sucrose transporter (SUT)—specifically, *SUT5*—and belongs to the wheat sucrose transporter group [32,52]. Previous reports showed that the *OsSUT* gene family controls transport of vacuolar reserve sucrose in rice, thereby effecting grain size, grain number, plant height, and final grain yield [53,54,55]. In wheat, the SUT family includes fourteen homologous genes, and gene expression analysis has shown that *TaSUT1* is highly expressed in developing grains and is significantly correlated with GWT, TGW, GN and grain size under drought [52,56]. In another study, the *TaSUT5* or *TraesCS2D02G505700/SUT5* gene was as identified as a hub gene, co-expression of which is associated with sink-to-source transition, to load sugars into the phloem of the wheat spike under low-light stress [57]. The promising candidate *TraesCS3A02G400500* was identified as a potential candidate gene within the *TaGL3AQTL* region and was functionally associated with TaLETM/LETM proteins [32]. In Arabidopsis, LETM proteins are important for mitochondrial protein accumulation, and loss of LETM function affects growth and can cause seed abortion, providing biological relevance to plant development [58]. Based on RNA seq data, *TraesCS3A02G400500* was found to be highly expressed in wheat grain and embryo [35,36,37]. Given the reported roles of LETM proteins in mitochondrial function and seed development, this gene represents a plausible candidate for further investigation. Importantly, all available wheat gene discovery and expression tools have highlighted high expression levels of *TraesCS2D02G505700* and *TraesCS3A02G400500* in grains [31,32,35,36,37]. For instance, the candidate gene *TraesCS2D02G505700* showed abundant expression in first leaf sheath, upper peduncle, and grain, in early, middle, and late plant development stages, respectively [35,36]. The expression pattern of the candidate gene *TraesCS3A02G400500* was similar where it was predominantly expressed in the first leaf sheath in early plant development stages, with spikes in middle development, slowly moving to grains in late physiological developmental stages [35,36]. These all support the hypothesis that *TraesCS2D02G505700* and *TraesCS3A02G400500* are primary gene candidates for *TaGL2DMTA/TaGL2DQTL* and *TaGL3AQTL*, respectively. However, further gene-level research study, considering recent advances in single-cell and spatial omics approaches, is required to prove their functional validation [59].

## 4. Materials and Methods

### 4.1. Plant Materials

The study involved a diversity panel (DP) of spring wheat and a biparental segregating population (SP). The DP comprised 153 elite spring wheat accessions, collectively representing a wide range of genetic backgrounds from Central Asia (Table S1). The SP was developed through crossing of the Kazakh commercial bread wheat cultivar ‘Kazakhstanskaya Rannespelaya’ with the United Kingdom reference cultivar ‘Paragon’. This cross generated 67 recombinant inbred lines (RILs), and these were genetically standardized through single-seed descent (SSD). Advancement of the SP to the F6 generation was performed in the glasshouse. Subsequent progression and homogenization from the F6 to F8 generations were carried out under speed breeding, using the protocol by Yermekbayev et al. (2026) [60]. The cultivar ‘Kazakhstanskaya Rannespelaya’ is widely grown in the country but has not been subjected to detailed genetic studies. The UK reference cultivar ‘Paragon’, by contrast, is well characterized genomically, and provides a complementary genetic background for QTL mapping [26,61,62,63].

### 4.2. Field Experimentation and Digital Grain Phenotyping

DP and SP were evaluated separately in experimental fields of the Kazakh Research Institute of Agriculture and Plant Growing (KRIAPG) in the Almaty region over the two wheat growing seasons of 2024 and 2025. The field experiments were designed using an incomplete block design. Twenty-five seeds of each genotype were sown per row, with two replications per genotype. The rows were spaced 20 cm apart and systematically organized into blocks, each containing 10 genotypes. Each replicate was independently randomized. Harvested spikes were hand threshed. Grain morphometric traits were evaluated using the MARVIN seed digital phenotyping system (MARViTECH GmbH, Wittenburg, Germany).

### 4.3. Statistical Analysis

Outliers were detected and removed by Tukey’s method. We tested for homogeneity of variances based on Bartlett’s test. One-way ANOVA and paired t-tests were employed to identify significant differences within and between the variables. Pearson’s method was used to calculate correlations between seasons and between grain-size-related phenotypes. We then estimated variance components for GL in META-R with a linear mixed model in lme4, using plot-level data and treating genotype as random. Entry-mean heritability was H^2^ = σ^2^G / (σ^2^G + σ^2^ε/r), where r is the number of replicates (r = 2 seasons) [64].

### 4.4. Genetic Diversity

The population genetic structure of the Central Asian wheat diversity panel was assessed using the widely used clustering software ‘STRUCTURE’ [65]. The number of hypothetical subpopulations (K) was examined over the range from K = 1 to K = 10, using the admixture model with correlated allele frequencies. For each K value, 5 independent runs were conducted to ensure reliable, consistent results. Each run included a burn-in of 100 iterations, followed by a 1000-iteration Markov chain Monte Carlo (MCMC) replication phase to achieve convergence. The most probable number of subpopulations was identified through the ΔK method [66]. To conduct a detailed investigation into the genetic relationships among various genotypes, a principal component analysis (PCA) was undertaken utilizing the GAPIT (Genome Association and Prediction Integrated Tool) package [67,68], as implemented in R. This analysis was performed using the genotype marker matrix to visualize population stratification and identify clustering patterns among the accessions.

### 4.5. Genotyping

High-quality genomic DNA was extracted from DP and SP plants using the Oktopure platform in accordance with the manufacturer’s protocol. Before genotyping, the concentration and quality of the DNA were assessed. Genotyping of the SP was conducted utilizing the 43K TaNGv1.1 SNP array on the Affymetrix Axiom platform [29]. The DP was genotyped using the Axiom 35K SNP array [28], which provides comprehensive genome-wide coverage of single-nucleotide polymorphisms (SNPs). Genotype calling and initial data processing were executed following the established standard array pipeline.

Quality control (QC) of genetic data for the DP was conducted to ensure the integrity of subsequent analyses. Individuals and markers exhibiting greater than 20% missing data were excluded from GWAS. SNPs with a minor allele frequency (MAF) below 0.05 were removed to eliminate low-frequency variants that would provide limited statistical power. Furthermore, markers displaying heterozygosity rates exceeding 15% were excluded. Missing genotype data were addressed using a combination of masking and imputation, with imputation being performed using the linkage disequilibrium k-nearest neighbor (LD-KNNi) method in TASSEL (v5.2.73) [69]. This method capitalizes on local linkage disequilibrium patterns and the genetic similarity among individuals to infer missing genotypes, thereby enhancing marker density and the overall completeness of the dataset.

For the SP, additional QC measures were applied to genetic data before QTL analyses. Highly polymorphic markers, as identified through the Axiom Analysis Suite, were retained. Monomorphic markers and those exhibiting greater than 20% missing data were excluded. Additional filtering steps involved removing duplicate markers (those with identical genotype patterns), redundant lines (RILs sharing identical genotypes), markers exhibiting significant segregation distortion (markers with an allele frequency below 0.10 or above 0.90 were removed), and markers lacking defined physical positions. The curated marker dataset served as the basis for QTL analyses using the R/qtl2 package in R [27]. Markers were ordered and assigned to chromosomes by their physical positions in IWGSC RefSeq v2.1, so the map was a physical marker map with positions in Mb.

### 4.6. Genome-Wide Association Study

Marker–trait associations (MTAs) were estimated using three models implemented in the GAPIT package v4.1 [67,68], including the Bayesian-information and Linkage-disequilibrium Iteratively Nested Key pathway (BLINK) [70], the Fixed and random model Circulating Probability Unification (FarmCPU) [71], and the Multiple Locus Mixed Model (MLMM) [72]. BLINK iteratively identifies pseudo-QTNs as covariates to improve the accuracy and power of marker–trait association detection [70]. FarmCPU separates fixed and random effects to reduce false positives while maintaining sensitivity [71]. MLMM extends the standard mixed model by including multiple markers simultaneously as cofactors, controlling for population structure and relatedness [72]. Each model was run separately, using means of replications across each year as the phenotype. All models included the first five principal components as fixed covariates (PCA.total = 5), and all other parameters followed the default parameters provided in GAPIT v4.1. No separate kinship matrix was specified, and model selection was not used [67,68]. The significance of associations between markers and traits was assessed based on a nominal significance threshold of *p* ≤ 0.001 (−log_10_(*p*) ≥ 3) (doi.org/10.3389/fpls.2021.820761, doi.org/10.3389/fgene.2021.661742) [73].

### 4.7. Linkage Mapping

The QTL analysis for the SP was performed utilizing the R/qtl2 package in the R environment [27]. Genotype probabilities were computed through application of the “calc_genoprob()” function with a genotyping error probability of 0.02, subsequently converted to allele probabilities using the “genoprob_to_alleleprob()” function. To address population structure and relatedness, a kinship matrix was estimated via the “calc_kinship()” function using the leave-one-chromosome-out (LOCO) method and incorporated into the ensuing analyses [74]. Genome-wide scans were conducted using a linear mixed model implemented in the “scan1()” function, with allele probabilities, phenotypic data, and the kinship matrix as inputs. The phenotype supplied to the scan was yearly means. Putative QTLs were identified utilizing the “find_peaks()” function, with loci exhibiting a logarithm of odds (LOD) threshold greater than 3.0 [25]. QTL support intervals were defined by a 1.5-LOD drop from the peak (drop = 1.5) and reported as Mb values between flanking markers.

### 4.8. Effective Allele Analysis

To assess the distribution of favorable alleles for grain length in DP and SP, the numbers of effective alleles at the significant SNP markers for each sample were calculated. Genotype data were taken from the HapMap genotype matrix in the IUPAC single-letter code format, with homozygous genotypes designated by a single nucleotide (C, G, T) and heterozygous genotypes by IUPAC ambiguity codes (S for C/G, Y for T/C). Samples were classified into three groups based on their genotype at this locus, such as homozygous for the effective allele (CC, 2 effective alleles), heterozygous (CT, 1 effective allele), and homozygous for the non-effective allele (TT, 0 effective alleles). The total number of samples in each group was summed and visualized as a histogram using the ggplot2 package in R software (version 4.6.1).

### 4.9. KASP Marker Design and Validation

KASP markers were developed for significant peak SNPs identified from both QTL analysis and GWAS. We designed primers by taking the 101 bp flanking each SNP from the IWGSC RefSeq v2.1 reference. Each assay had two allele-specific forward primers that differed at the 3′ terminal base and had one common reverse primer.

Each allele-specific forward primer carried a 5′ tail matching one reporter of the KASP master mix: the FAM tail (5′-GAAGGTGACCAAGTTCATGCT-3′) and HEX tail (5′-GAAGGTCGGAGTCAACGGATT-3′). We prepared the assay mix from 12 µM of each allele-specific primer and 30 µM of the common primer. Each 10 µL reaction contained 5 µL 2× KASP Master Mix, 0.11 µL assay mix, 2 µL genomic DNA, and 2.89 µL of nuclease-free water. We extracted DNA from leaf tissue by the CTAB method, and measured its concentration with a NanoDrop spectrophotometer (Thermo Fisher Scientific, Waltham, MA, USA).

We ran reactions in 96-well plates on a Bio-Rad CFX96 real-time PCR system (Bio-Rad Laboratories, Hercules, CA, USA). The program started with 15 min at 95 °C, followed by 11 touchdown cycles of 20 s at 95 °C and 60 s at 65 °C, with the annealing temperature falling by 0.8 °C per cycle to 57 °C. Thirty-one further cycles of 20 s at 94 °C and 60 s at 57 °C followed. We read endpoint fluorescence in the FAM and HEX channels at 37 °C for 1 min.

We called genotypes in Bio-Rad CFX Maestro (Version 4.1) from the FAM–HEX allelic discrimination plot. Each plate included 2 no-template controls.

Developed KASP markers segregating with the trait of interest were validated on DP. Then, genotype–phenotype association for grain length was evaluated.

### 4.10. Functional Gene Annotation

To identify the physical locations of significant peak SNPs, these were mapped against the wheat reference genome ‘Chinese Spring’ [30]. We searched for candidate genes within a sliding window of 10 kb up and downstream from the significant SNP marker until identification of the first candidate gene. We further characterized each candidate gene based on its molecular function, tissue-specific expression, and reported associations with GL, using open-source evidence-based gene discovery and expression resources such as expVIP, Wheat eFP Browser, KnetMiner, Enseml Plants and WheatOmics [31,32,33,35,36,37]. Expression profiles were presented only for the most promising candidate genes.

## 5. Conclusions

Larger grains are an important breeding target for wheat breeders, producers, and consumers in Central Asia. In this study, we identified genomic regions associated with grain length in Central Asian wheat germplasm, providing new insights into the genetic basis of this trait in the region. However, the available data did not reveal detectable genetic or phenotypic variation in grain width within the studied panels. The contrasting patterns of variation indicate that GL and GW may be influenced by distinct genetic factors in this germplasm. Grain length was positively correlated with grain weight and thousand-grain weight, highlighting its potential contribution to grain weight. Furthermore, chromosomes 2DL and 3AL emerged as promising targets for marker-assisted selection to improve grain morphology in Central Asian wheat. The KASP markers developed for *TaGL2DMTA*, *TaGL2DQTL* and *TaGL3AQTL* loci provide practical tools for identifying favorable alleles and supporting their use in wheat breeding.

## Figures and Tables

**Table 1:** Genetic parameters for GL based on BLUP values in DP and SP. Significance codes: *** *p* < 0.001.

Group	Growing Season	Mean	SD	Range	CV (%)	H^2^	Genotype
**DP**	2024	6.12	0.29	5.24–6.75	4.69	0.91	***
	2025	6.22	0.28	5.28–6.84	4.49		
**SP**	2024	5.73	0.25	5.17–6.28	4.36	0.93	***
	2025	5.94	0.23	5.23–6.55	3.93		

**Table 2:** Estimation of the optimal number of subpopulations based on structure analysis using the Evanno method. Significance codes: * *p* < 0.05.

K	Rep	Mean LnP(K)	SD LnP(K)	Ln′(K)	Ln″(K)	Delta K
1	5	−3,488,423.08	142.54	NA	NA	NA
2 *	5	−3,153,277.38	27,534.54	335,145.7	27,715,777.4	1006.58
3	5	−30,533,909.04	58,180,940.4	−27,380,632	27,067,937.4	0.47
4	5	−30,846,603.3	31,340,110.7	−312,694.26	184,207,813	5.88
5	5	−215,367,111	181,245,933	−184,520,508	155,851,733	0.86
6	5	−555,739,352.2	531,443,581	−340,372,241	409,380,958	0.77
7	5	−1,305,492,551	548,298,671	−749,753,199	1,337,156,418	2.44
8	5	−3,392,402,168	1,676,616,819	−208,690,961	1,451,618,237	0.87
9	5	−4,027,693,547	2,979,918,952	−635,291,379	147,788,063	0.05
10	5	−4,810,772,990	3,658,957,572	−783,079,443	NA	NA

**Table 3:** MTAs for GL in the DP, detected by BLINK, FarmCPU, and MLMM, for the 2024 and 2025 growing seasons. Models lists the models that detected the MTA, with the *p* value being marked by asterisks after each name (*** *p* < 0.001, **** *p* < 0.0001). Position is given in bp. LOG10p (−log10 *p*) and Effect (mm) are means across the models.

Chromosome	SNP ID	Growing Seasons	Models	Position	LOG10*p*	Effect
1A	AX–94390433	2025	BLINK ****	58,751,720	6.855	0.354
	AX–94465963	2025	MLMM ***	574,498,568	3.,235	0.104
	AX–95164006	2025	MLMM ***	575,245,269	3.092	0.103
1B	AX–94874112	2024	BLINK ***	10,204,574	3.08	0.136
	AX–94546205	2024–2025	BLINK ****, FarmCPU ****, MLMM ***	37,727,756	5.095	0.186
	AX–94527502	2024–2025	FarmCPU ****, BLINK ****	670,432,537	6.581	0.195
1D	AX–94771995	2025	FarmCPU ****	10,0124	6.271	0.328
	AX–94522381	2024	FarmCPU ***	5,453,084	3.766	0.18
	AX–94694366	2024	MLMM ***	11,664,706	3.172	−0.128
2B	AX–95003568	2025	MLMM ***	9,620,774	3.195	0.225
	AX–94555593	2024–2025	FarmCPU ****, BLINK ***	32,964,020	4.476	−0.141
	AX–95141885	2024–2025	BLINK ****, MLMM ***	546,426,927	3.923	0.452
	AX–94478727	2024	FarmCPU ****	713,697,641	6.019	−0.192
2D	AX–95178106	2024	BLINK ****	519,133,455	4.073	0.34
	AX–94457710	2024–2025	BLINK ****, FarmCPU ****, MLMM ***	599,946,636	5.784	0.317
3A	AX–94492007	2024	BLINK ****	41,487,705	3.825	0.119
	AX–94525583	2024	BLINK ****	717,653,782	4.302	0.174
	AX–94660817	2024	BLINK ****	717,903,279	4.143	0.104
3B	AX–95232967	2024	BLINK ****, FarmCPU ****	730,311,614	8.728	0.175
	AX–95012984	2024	MLMM ****	736,711,881	5.042	0.163
	AX–94916747	2025	FarmCPU ****, MLMM ***	820,791,977	4.444	0.108
3D	AX–94885021	2024	BLINK****	588,999,605	4.018	0.193
4B	AX–94425133	2024	BLINK***	535,085,299	3.02	0.172
5A	AX–94955528	2025	BLINK****, FarmCPU****	476,602,451	5.122	0.228
	AX–95199834	2024	BLINK ***	679,757,426	3.142	0.154
5B	AX–94943796	2024	BLINK ***	13,725,620	3.623	0.197
	AX–95259052	2025	MLMM ***	143,718,090	3.419	0.238
	AX–94912015	2024	BLINK ***	546,826,568	3.519	0.115
5D	AX–94754558	2024	MLMM ***	43,408,872	3.124	−0.146
	AX–95008379	2024	BLINK ***	84,317,507	3.094	0.157
6A	AX–94583437	2025	MLMM ***	615,130,202	3.035	0.143
6B	AX–94527004	2024	BLINK ****	669,025,908	6.12	−0.108
7A	AX–95172836	2024	MLMM ***	667,336,533	3.059	0.542
	AX–94722616	2025	BLINK ****, FarmCPU ****	677,847,304	9.223	0.261
7B	AX–94676649	2025	FarmCPU ***	674,556,443	3.158	−0.145
	AX–95012310	2025	MLMM ***	741,571,274	3.843	−0.136
7D	AX–94541307	2025	MLMM ***	38,735,794	3.555	−0.156

**Table 4:** QTL for GL detected in the SP for the 2024 and 2025 growing seasons. PVE—phenotypic variance explained; MAF—minor allele frequency; Pos—physical position (bp); LOD—log10 of the likelihood ratio; Effect—additive effect (mm); Donor—parent that contributed the favorable allele (KZR, Kazakhstanskaya Rannespelaya; PAR, Paragon). ***—*p* < 0.001.

Chromosome	SNP ID	Growing Season	PVE, %	Significance	MAF	Pos	LOG10(*p*)	Effect	Donor
3A	AX–643825530	2024	18.27	***	0.57	647,002,945	4.779	0.26	KZR
2D	AX–643815838	2025	24.36	***	0.52	422,890,054	3.222	0.13	PAR
3A	AX–643825530	2025	28.22	***	0.57	647,002,945	4.037	0.21	KZR

**Table 5:** List of trait-associated KASP markers.

Marker ID	Chromosome	Position	SNP	Dye	Sequence	Source
AX–94457710	2D	599,946,636	C/T	FAM	GAAGGTGACCAAGTTCATGCTCCTGAATAACTCATTGTACTCTT	GWAS
				HEX	GAAGGTCGGAGTCAACGGATTCCTGAATAACTCATTGTACTCTC	
				Common	TACTTTGTCGGGCAAGAGCT	
AX–643815838	2D	422,890,054	T/C	FAM	GAAGGTGACCAAGTTCATGCTTTGAACTGCATGGAAGGTGAGC	QTL
				HEX	GAAGGTCGGAGTCAACGGATTTTGAACTGCATGGAAGGTGAGT	
				Common	TCCTCCTCTGCGCTTCTTCT	
AX–643825530	3A	647,002,945	C/G	FAM	GAAGGTGACCAAGTTCATGCTAACAGCGATTCTTGGCCTGTCAC	QTL
				HEX	GAAGGTCGGAGTCAACGGATTAACAGCGATTCTTGGCCTGTCAG	
				Common	CATCCAAGTCTTGACATTTACGGT	

## Data Availability

The data presented in this study are available in the article. The raw data are available on request from the corresponding author.

## Supplementary Materials

The following supporting information can be downloaded at: https://www.mdpi.com/article/10.3390/plants15213361/s1, Table S1: List of genotypes.

## References

[1] Shiferaw B., Smale M., Braun H.J., Duveiller E., Reynolds M., Muricho G. (2013). Crops That Feed the World 10. Past Successes and Future Challenges to the Role Played by Wheat in Global Food Security. Food Secur..

[2] Savary S., Willocquet L., Pethybridge S.J., Esker P., McRoberts N., Nelson A. (2019). The Global Burden of Pathogens and Pests on Major Food Crops. Nat. Ecol. Evol..

[3] Romanovska P., Schauberger B., Gornott C. (2023). Wheat Yields in Kazakhstan Can Successfully Be Forecasted Using a Statistical Crop Model. Eur. J. Agron..

[4] Amalova A., Griffiths S., Abugalieva S., Turuspekov Y. (2024). Genome-Wide Association Study of Yield-Related Traits in a Nested Association Mapping Population Grown in Kazakhstan. Agronomy.

[5] Zhao J., Sun L., Gao H., Hu M., Mu L., Cheng X., Wang J., Zhao Y., Li Q., Wang P. (2023). Genome-Wide Association Study of Yield-Related Traits in Common Wheat (*Triticum aestivum* L.) under Normal and Drought Treatment Conditions. Front. Plant Sci..

[6] Turuspekov Y., Baibulatova A., Yermekbayev K., Tokhetova L., Chudinov V., Sereda G., Ganal M., Griffiths S., Abugalieva S. (2017). GWAS for Plant Growth Stages and Yield Components in Spring Wheat (*Triticum aestivum* L.) Harvested in Three Regions of Kazakhstan. BMC Plant Biol..

[7] Hu J., Wang X., Zhang G., Jiang P., Chen W., Hao Y., Ma X., Xu S., Jia J., Kong L. (2020). QTL Mapping for Yield-Related Traits in Wheat Based on Four RIL Populations. Theor. Appl. Genet..

[8] Yang L., Zhao D., Meng Z., Xu K., Yan J., Xia X., Cao S., Tian Y., He Z., Zhang Y. (2020). QTL Mapping for Grain Yield-Related Traits in Bread Wheat via SNP-Based Selective Genotyping. Theor. Appl. Genet..

[9] Liu W., Naruoka Y., Miller K., Garland-Campbell K.A., Carter A.H. (2018). Characterizing and Validating Stripe Rust Resistance Loci in US Pacific Northwest Winter Wheat Accessions (*Triticum aestivum* L.) by Genome-Wide Association and Linkage Mapping. Plant Genome.

[10] Gegas V.C., Nazari A., Griffiths S., Simmonds J., Fish L., Orford S., Sayers L., Doonan J.H., Snape J.W. (2010). A Genetic Framework for Grain Size and Shape Variation in Wheat. Plant Cell.

[11] Breseghello F., Sorrells M.E. (2007). QTL Analysis of Kernel Size and Shape in Two Hexaploid Wheat Mapping Populations. Field Crops Res..

[12] Tekeu H., Ngonkeu E.L.M., Bélanger S., Djocgoué P.F., Abed A., Torkamaneh D., Boyle B., Tsimi P.M., Tadesse W., Jean M. (2021). GWAS Identifies an Ortholog of the Rice D11 Gene as a Candidate Gene for Grain Size in an International Collection of Hexaploid Wheat. Sci. Rep..

[13] Rasheed A., Xia X., Ogbonnaya F., Mahmood T., Zhang Z., Mujeeb-Kazi A., He Z. (2014). Genome-Wide Association for Grain Morphology in Synthetic Hexaploid Wheats Using Digital Imaging Analysis. BMC Plant Biol..

[14] Philipp N., Weichert H., Bohra U., Weschke W., Schulthess A.W., Weber H. (2018). Grain Number and Grain Yield Distribution along the Spike Remain Stable despite Breeding for High Yield in Winter Wheat. PLoS ONE.

[15] Kumar A., Mantovani E.E., Seetan R., Soltani A., Echeverry-Solarte M., Jain S., Simsek S., Doehlert D., Alamri M.S., Elias E.M. (2016). Dissection of Genetic Factors Underlying Wheat Kernel Shape and Size in an Elite × Nonadapted Cross Using a High Density SNP Linkage Map. Plant Genome.

[16] Evers T., Millar S. (2002). Cereal Grain Structure and Development: Some Implications for Quality. J. Cereal Sci..

[17] Ma L., Li T., Hao C., Wang Y., Chen X., Zhang X. (2016). TaGS5-3A, a Grain Size Gene Selected during Wheat Improvement for Larger Kernel and Yield. Plant Biotechnol. J..

[18] Yu H., Hao Y., Li M., Dong L., Che N., Wang L., Song S., Liu Y., Kong L., Shi S. (2022). Genetic Architecture and Candidate Gene Identification for Grain Size in Bread Wheat by GWAS. Front. Plant Sci..

[19] Wang X., Dong L., Hu J., Pang Y., Hu L., Xiao G., Ma X., Kong X., Jia J., Wang H. (2019). Dissecting Genetic Loci Affecting Grain Morphological Traits to Improve Grain Weight via Nested Association Mapping. Theor. Appl. Genet..

[20] El Baouchi A., Ibriz M., Dreisigacker S., Lopes M.S., Sanchez-Garcia M. (2024). Dissection of the Genetic Basis of Genotype by Environment Interactions for Morphological Traits and Protein Content in Winter Wheat Panel Grown in Morocco and Spain. Plants.

[21] Hong Y., Zhang M., Yuan Z., Zhu J., Lv C., Guo B., Wang F., Xu R. (2024). Genome-Wide Association Studies Reveal Stable Loci for Wheat Grain Size under Different Sowing Dates. PeerJ.

[22] Ma J., Liu Y., Zhang P., Chen T., Tian T., Wang P., Che Z., Shahinnia F., Yang D. (2022). Identification of Quantitative Trait Loci (QTL) and Meta-QTL Analysis for Kernel Size-Related Traits in Wheat (*Triticum aestivum* L.). BMC Plant Biol..

[23] Chen Z., Cheng X., Chai L., Wang Z., Bian R., Li J., Zhao A., Xin M., Guo W., Hu Z. (2020). Dissection of Genetic Factors Underlying Grain Size and Fine Mapping of QTgw.Cau-7D in Common Wheat (*Triticum aestivum* L.). Theor. Appl. Genet..

[24] Wu Q.H., Chen Y.X., Zhou S.H., Fu L., Chen J.J., Xiao Y., Zhang D., Ouyang S.H., Zhao X.J., Cui Y. (2015). High-Density Genetic Linkage Map Construction and QTL Mapping of Grain Shape and Size in the Wheat Population Yanda1817 × Beinong6. PLoS ONE.

[25] Cao J., Shang Y., Xu D., Xu K., Cheng X., Pan X., Liu X., Liu M., Gao C., Yan S. (2020). Identification and Validation of New Stable QTLs for Grain Weight and Size by Multiple Mapping Models in Common Wheat. Front. Genet..

[26] Amalova A., Griffiths S., Abugalieva A., Abugalieva S., Turuspekov Y. (2025). Identification of Quantitative Trait Loci for Grain Quality Traits in a Pamyati Azieva × Paragon Bread Wheat Mapping Population Grown in Kazakhstan. Plants.

[27] Broman K.W., Gatti D.M., Simecek P., Furlotte N.A., Prins P., Sen Ś., Yandell B.S., Churchill G.A. (2019). R/Qtl2: Software for Mapping Quantitative Trait Loci with High-Dimensional Data and Multiparent Populations. Genetics.

[28] Allen A.M., Winfield M.O., Burridge A.J., Downie R.C., Benbow H.R., Barker G.L.A., Wilkinson P.A., Coghill J., Waterfall C., Davassi A. (2017). Characterization of a Wheat Breeders’ Array Suitable for High-Throughput SNP Genotyping of Global Accessions of Hexaploid Bread Wheat (*Triticum aestivum*). Plant Biotechnol. J..

[29] Burridge A.J., Winfield M., Przewieslik-Allen A., Edwards K.J., Siddique I., Barral-Arca R., Griffiths S., Cheng S., Huang Z., Feng C. (2024). Development of a next Generation SNP Genotyping Array for Wheat. Plant Biotechnol. J..

[30] Appels R., Eversole K., Feuillet C., Keller B., Rogers J., Stein N., Pozniak C.J., Choulet F., Distelfeld A., Poland J. (2018). Shifting the Limits in Wheat Research and Breeding Using a Fully Annotated Reference Genome. Science.

[31] Ma S., Wang M., Wu J., Guo W., Chen Y., Li G., Wang Y., Shi W., Xia G., Fu D. (2021). WheatOmics: A Platform Combining Multiple Omics Data to Accelerate Functional Genomics Studies in Wheat. Mol. Plant.

[32] Hassani-Pak K., Singh A., Brandizi M., Hearnshaw J., Parsons J.D., Amberkar S., Phillips A.L., Doonan J.H., Rawlings C. (2021). KnetMiner: A Comprehensive Approach for Supporting Evidence-Based Gene Discovery and Complex Trait Analysis across Species. Plant Biotechnol. J..

[33] Yates A.D., Austine-Orimoloye O., Azov A.G., Barba M., Barnes I., Barrera-Enriquez V.P., Becker A., Bennett R., Berry A., Bhai J. (2026). Ensembl 2026. Nucleic Acids Res..

[34] Kinsella R.J., Kähäri A., Haider S., Zamora J., Proctor G., Spudich G., Almeida-King J., Staines D., Derwent P., Kerhornou A. (2011). Ensembl BioMarts: A Hub for Data Retrieval across Taxonomic Space. Database.

[35] Ramírez-González R.H., Borrill P., Lang D., Harrington S.A., Brinton J., Venturini L., Davey M., Jacobs J., Van Ex F., Pasha A. (2018). The Transcriptional Landscape of Polyploid Wheat. Science.

[36] Winter D., Vinegar B., Nahal H., Ammar R., Wilson G.V., Provart N.J. (2007). An “Electronic Fluorescent Pictograph” Browser for Exploring and Analyzing Large-Scale Biological Data Sets. PLoS ONE.

[37] Borrill P., Ramirez-Gonzalez R., Uauy C. (2016). ExpVIP: A Customizable RNA-Seq Data Analysis and Visualization Platform. Plant Physiol..

[38] Li T., Li Q., Wang J., Yang Z., Tang Y., Su Y., Zhang J., Qiu X., Pu X., Pan Z. (2022). High-Resolution Detection of Quantitative Trait Loci for Seven Important Yield-Related Traits in Wheat (*Triticum aestivum* L.) Using a High-Density SLAF-Seq Genetic Map. BMC Genom. Data.

[39] Brinton J., Simmonds J., Minter F., Leverington-Waite M., Snape J., Uauy C. (2017). Increased Pericarp Cell Length Underlies a Major Quantitative Trait Locus for Grain Weight in Hexaploid Wheat. New Phytol..

[40] Xie Q., Mayes S., Sparkes D.L. (2015). Carpel Size, Grain Filling, and Morphology Determine Individual Grain Weight in Wheat. J. Exp. Bot..

[41] Morgounov A.I., Braun H.J., Ketata H., Paroda R. (2005). International Cooperation for Winter Wheat Improvement in Central Asia: Results and Perspectives. Turk. J. Agric. For..

[42] Amalova A., Abugalieva S., Babkenov A., Babkenova S., Turuspekov Y. (2021). Genome-Wide Association Study of Yield Components in Spring Wheat Collection Harvested under Two Water Regimes in Northern Kazakhstan. PeerJ.

[43] Zhao Z., Rebetzke G.J., Zheng B., Chapman S.C., Wang E. (2019). Modelling Impact of Early Vigour on Wheat Yield in Dryland Regions. J. Exp. Bot..

[44] Jiang C., Xu Z., Fan X., Zhou Q., Ji G., Liao S., Wang Y., Ma F., Zhao Y., Wang T. (2023). Genetic Dissection of Major QTL for Grain Number per Spike on Chromosomes 5A and 6A in Bread Wheat (*Triticum aestivum* L.). Front. Plant Sci..

[45] Ramya P., Chaubal A., Kulkarni K., Gupta L., Kadoo N., Dhaliwal H.S., Chhuneja P., Lagu M., Gupta V. (2010). *QTL Mapping of* 1000-Kernel Weight, Kernel Length, and Kernel Width in Bread Wheat (*Triticum aestivum* L.). J. Appl. Genet..

[46] Yan L., Liang F., Xu H., Zhang X., Zhai H., Sun Q., Ni Z. (2017). Identification of QTL for Grain Size and Shape on the D Genome of Natural and Synthetic Allohexaploid Wheats with Near-Identical AABB Genomes. Front. Plant Sci..

[47] Su Q., Zhang X., Zhang W., Zhang N., Song L., Liu L., Xue X., Liu G., Liu J., Meng D. (2018). QTL Detection for Kernel Size and Weight in Bread Wheat (*Triticum aestivum* L.) Using a High-Density SNP and SSR-Based Linkage Map. Front. Plant Sci..

[48] Li S., Wang L., Meng Y., Hao Y., Xu H., Hao M., Lan S., Zhang Y., Lv L., Zhang K. (2021). Dissection of Genetic Basis Underpinning Kernel Weight-Related Traits in Common Wheat. Plants.

[49] Lyu J., Wang D., Sun N., Yang F., Li X., Mu J., Zhou R., Zheng G., Yang X., Zhang C. (2024). The TaSnRK1-TabHLH489 Module Integrates Brassinosteroid and Sugar Signalling to Regulate the Grain Length in Bread Wheat. Plant Biotechnol. J..

[50] Rustgi S., Shafqat M.N., Kumar N., Baenziger P.S., Ali M.L., Dweikat I., Campbell B.T., Gill K.S. (2013). Genetic Dissection of Yield and Its Component Traits Using High-Density Composite Map of Wheat Chromosome 3A: Bridging Gaps between QTLs and Underlying Genes. PLoS ONE.

[51] Jadon V., Gajghate R., Devate N.B., Amaresh, Krishna H., Krishnappa G., Dosad S., Jain N., Singh P.K., Singh G.P. (2026). High Resolution Mapping of Agro-Morphological and Grain Traits in Bread Wheat Using SNP-Based QTL Analysis. PLoS ONE.

[52] Al-Sheikh Ahmed S., Zhang J., Ma W., Dell B. (2018). Contributions of TaSUTs to Grain Weight in Wheat under Drought. Plant Mol. Biol..

[53] Sun C., Wang Y., Yang X., Tang L., Wan C., Liu J., Chen C., Zhang H., He C., Liu C. (2023). MATE Transporter GFD1 Cooperates with Sugar Transporters, Mediates Carbohydrate Partitioning and Controls Grain-Filling Duration, Grain Size and Number in Rice. Plant Biotechnol. J..

[54] Yang M.Y., Yang X., Yan Z., Chao Q., Shen J., Shui G.H., Guo P.M., Wang B.C. (2023). OsTST1, a Key Tonoplast Sugar Transporter from Source to Sink, Plays Essential Roles in Affecting Yields and Height of Rice (*Oryza sativa* L.). Planta.

[55] Eom J.S., Cho J.I., Reinders A., Lee S.W., Yoo Y., Tuan P.Q., Choi S.B., Bang G., Park Y.I., Cho M.H. (2011). Impaired Function of the Tonoplast-Localized Sucrose Transporter in Rice, OsSUT2, Limits the Transport of Vacuolar Reserve Sucrose and Affects Plant Growth. Plant Physiol..

[56] Hou Q., Gao J., Qin Z., Sun H., Wang H., Yuan S., Zhang F., Yang W. (2024). Genome-Wide Identification and Expression Analysis of Sucrose Transporter Gene Family in Wheat Lines under Heat Stress. Agronomy.

[57] Yang H., Li Y., Qiao Y., Sun H., Liu W., Qiao W., Li W., Liu M., Dong B. (2023). Low Light Stress Promotes New Tiller Regeneration by Changing Source–Sink Relationship and Activating Expression of Expansin Genes in Wheat. Plant Cell Environ..

[58] Zhang B., Carrie C., Ivanova A., Narsai R., Murcha M.W., Duncan O., Wang Y., Law S.R., Albrecht V., Pogson B. (2012). LETM Proteins Play a Role in the Accumulation of Mitochondrially Encoded Proteins in *Arabidopsis thaliana* and AtLETM2 Displays Parent of Origin Effects. J. Biol. Chem..

[59] Wang F., Wang F., Wu Y., Pu L., Le L. (2026). Single-Cell Insights into Plant Growth, Adaptation, and Evolution. J. Integr. Plant Biol..

[60] Yermekbayev K., Abduakassov A., Shoken M., Sartbayeva Z., Baimyrzayev K., Bauyrzhan M., Abugalieva S., Turuspekov Y., Mahmood Z. (2026). Establishment of the First Speed Breeding Facility in Central Asia: Development of an Accessible Bread Wheat (*Triticum aestivum* L.) Protocol for Resource-Limited Research Institutions. Front. Plant Sci..

[61] White B., Lux T., Rusholme-Pilcher R., Juhász A., Kaithakottil G., Duncan S., Simmonds J., Rees H., Wright J., Colmer J. (2025). De Novo Annotation Reveals Transcriptomic Complexity across the Hexaploid Wheat Pan-Genome. Nat. Commun..

[62] Yermekbayev K., Griffiths S., Chhetry M., Leverington-Waite M., Orford S., Amalova A., Abugalieva S., Turuspekov Y. (2020). Construction of a Genetic Map of RILs Derived from Wheat (*T. aestivum* L.) Varieties Pamyati Azieva × Paragon Using High-Throughput SNP Genotyping Platform KASP—Kompetitive Allele Specific PCR. Russ. J. Genet..

[63] Amalova A., Yermekbayev K., Griffiths S., Abugalieva S., Babkenov A., Fedorenko E., Abugalieva A., Turuspekov Y. (2022). Identification of Quantitative Trait Loci of Agronomic Traits in Bread Wheat Using a Pamyati Azieva × Paragon Mapping Population Harvested in Three Regions of Kazakhstan. PeerJ.

[64] Alvarado G., Rodríguez F.M., Pacheco A., Burgueño J., Crossa J., Vargas M., Pérez-Rodríguez P., Lopez-Cruz M.A. (2020). META-R: A Software to Analyze Data from Multi-Environment Plant Breeding Trials. Crop J..

[65] Evanno G., Regnaut S., Goudet J. (2005). Detecting the Number of Clusters of Individuals Using the Software STRUCTURE: A Simulation Study. Mol. Ecol..

[66] Pritchard J.K., Stephens M., Donnelly P. (2000). Inference of Population Structure Using Multilocus Genotype Data. Genetics.

[67] Wang J., Zhang Z. (2021). GAPIT Version 3: Boosting Power and Accuracy for Genomic Association and Prediction. Genom. Proteom. Bioinform..

[68] Lipka A.E., Tian F., Wang Q., Peiffer J., Li M., Bradbury P.J., Gore M.A., Buckler E.S., Zhang Z. (2012). GAPIT: Genome Association and Prediction Integrated Tool. Bioinformatics.

[69] Bradbury P.J., Zhang Z., Kroon D.E., Casstevens T.M., Ramdoss Y., Buckler E.S. (2007). TASSEL: Software for Association Mapping of Complex Traits in Diverse Samples. Bioinformatics.

[70] Huang M., Liu X., Zhou Y., Summers R.M., Zhang Z. (2019). BLINK: A Package for the next Level of Genome-Wide Association Studies with Both Individuals and Markers in the Millions. Gigascience.

[71] Liu X., Huang M., Fan B., Buckler E.S., Zhang Z. (2016). Iterative Usage of Fixed and Random Effect Models for Powerful and Efficient Genome-Wide Association Studies. PLoS Genet..

[72] Segura V., Vilhjálmsson B.J., Platt A., Korte A., Seren Ü., Long Q., Nordborg M. (2012). An Efficient Multi-Locus Mixed-Model Approach for Genome-Wide Association Studies in Structured Populations. Nat. Genet..

[73] Flint-Garcia S.A., Thornsberry J.M., Buckler E.S. (2003). Structure of Linkage Disequilibrium in Plants. Annu. Rev. Plant Biol..

[74] Yu J., Pressoir G., Briggs W.H., Vroh Bi I., Yamasaki M., Doebley J.F., McMullen M.D., Gaut B.S., Nielsen D.M., Holland J.B. (2006). A Unified Mixed-Model Method for Association Mapping That Accounts for Multiple Levels of Relatedness. Nat. Genet..

